# Maternal thyroid disorder in pregnancy and risk of cerebral palsy in the child: a population-based cohort study

**DOI:** 10.1186/s12887-018-1152-5

**Published:** 2018-05-31

**Authors:** Tanja Gram Petersen, Anne-Marie Nybo Andersen, Peter Uldall, Nigel Paneth, Ulla Feldt-Rasmussen, Mette Christophersen Tollånes, Katrine Strandberg-Larsen

**Affiliations:** 1 Section of Social Medicine, Department of Public Health, University of Copenhagen, Gothersgade 160, 1123 Copenhagen, Denmark; 2 Pediatric Department at Rigshospitalet, Juliane Maries Vej 8, 2100 Copenhagen, Denmark; 3 Department of Epidemiology & Biostatistics and Department of Pediatrics and Human Development, Michigan State University, 909 Fee Road, East Lansing, MI 48824 USA; 4 Department of Medical Endocrinology at Rigshospitalet, Ole Maaloees Vej 26, 2200 Copenhagen, Denmark; 5 Domain for Mental and Physical Health at Norwegian Institute of Public Health, Kalfarveien 31, 5020 Bergen, Norway

**Keywords:** Maternal thyroid disorder, Pregnancy, Prenatal exposure, Cerebral palsy, The Danish National Birth Cohort, The Norwegian mother and child cohort study, Register-based cohort

## Abstract

**Background:**

Cerebral palsy is the most frequent motor disability in childhood, but little is known about its etiology. It has been suggested that cerebral palsy risk may be increased by prenatal thyroid hormone disturbances. The objective of this study was to investigate whether maternal thyroid disorder is associated with increased risk of cerebral palsy.

**Methods:**

A population-based cohort study using two study populations. 1) 1,270,079 children born in Denmark 1979–2007 identified in nationwide registers, and 2) 192,918 children born 1996–2009 recruited into the Danish National Birth Cohort and The Norwegian Mother and Child Cohort study, combined in the MOthers and BAbies in Norway and Denmark (MOBAND) collaboration cohort. Register-based and self-reported information on maternal thyroid disorder was studied in relation to risk of cerebral palsy and its unilateral and bilateral spastic subtypes using multiple logistic regression. Children were followed from the age of 1 year to the age of 6 years, and cerebral palsy was identified in nationwide registers with verified diagnoses.

**Results:**

In register data, hypothyroidism was recognized in 12,929 (1.0%), hyperthyroidism in 9943 (0.8%), and unclassifiable thyroid disorder in 753 (< 0.1%) of the mothers. The odds ratio for an association between maternal thyroid disorder and bilateral spastic cerebral palsy was 1.0 (95% CI: 0.7–1.5). Maternal thyroid disorder identified during pregnancy was associated with elevated risk of unilateral spastic cerebral palsy (odds ratio 3.1 (95% CI: 1.2–8.4)). In MOBAND, 3042 (1.6%) of the mothers reported a thyroid disorder in pregnancy, which was not associated with cerebral palsy overall (odds ratio 1.2 (95% CI: 0.6–2.4)).

**Conclusions:**

Maternal thyroid disorder overall was not related to bilateral spastic cerebral palsy, but maternal thyroid disorder identified in pregnancy was associated with increased risk of unilateral spastic cerebral palsy. These findings should be replicated in studies making use of maternal blood samples.

**Electronic supplementary material:**

The online version of this article (10.1186/s12887-018-1152-5) contains supplementary material, which is available to authorized users.

## Background

Cerebral palsy (CP) is the most prevalent severe motor disability in childhood affecting approximately 2 per 1000 live-born children [1]. Recent studies have suggested that birth complications constitute only a small part of the factors contributing to the multifactorial etiology of CP, and that most CP risk factors probably operate prenatally [2]. Several studies have demonstrated that elevated maternal serum levels of thyrotropin and low thyroid hormone in pregnancy may affect child neurodevelopment, including motor function [3–6]. A number of studies have linked thyroid hormone disturbances of mothers or newborns to CP [7–11], but not every study finds the association [12].

Endogenous fetal thyroid hormone production begins around 10-18th week of gestation. The fetus, therefore, depends on maternal thyroid hormone entirely in early pregnancy, and from mid-gestation fetal thyroid hormone production acts in concert with the maternal hypothalamic-pituitary-thyroid axis [8, 13, 14]. Thyroid hormone is required for many aspects of brain development, including myelination of nerve cells, and insufficient myelination is often present in individuals with CP [8, 15]. Moreover, hypo- and hyperthyroidism are correlated with coagulation abnormalities that can lead to ischemia or bleeding, which may underlie unilateral spastic CP [16, 17]. Thyroid diseases are difficult to stabilize with treatment, especially in pregnancy [18], and it is not unusual for overtreatment to lead women diagnosed with hypothyroidism to have elevated thyroid hormone levels and women with hyperthyroidism to have reduced thyroid hormone levels. Thus, we aimed to investigate the association between maternal thyroid disorder in pregnancy and risk of cerebral palsy in the child in two study populations in Denmark and Norway, each one with a distinct research advantage. One study population permitted examination of different thyroid disorder and CP subtypes in a very large study sample, while the other provided opportunities for performing analyses that controlled for lifestyle factors.

## Methods

### Study design and populations (Fig. 1)

One study population is register-based and comprises children born in the eastern part of Denmark in 1979–1996, and children born in all of Denmark in 1997–2007. In total, 1,277,101 live births were identified through the Danish Civil Registration System [19]. Individual-level data from nationwide registers on the children and their mothers were linked by use of the unique personal identifier to which all live-born children in Denmark are assigned. We excluded infant deaths (*n* = 7022), leaving 1,270,079 children for the final analyses.

**Fig. 1:**
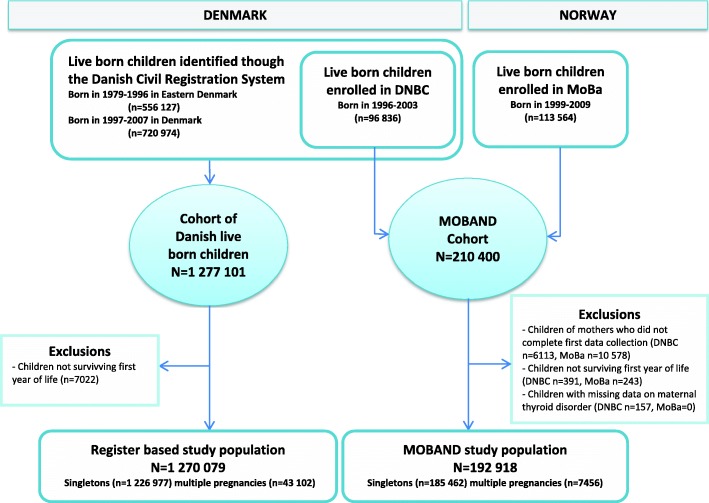
Flowchart of the register-based study population and MOBAND study population. Abbreviations: MOBAND, MOthers and BAbies in Norway and Denmark (MOBAND). DNBC, Danish National Birth Cohort. MoBa, Norwegian Mother and Child Cohort study

The other study population is derived from the MOthers and BAbies in Norway and Denmark (MOBAND) collaboration cohort [20], which consists of pooled data from the Danish National Birth Cohort (DNBC) and The Norwegian Mother and Child Cohort study (MoBa) [21, 22]. MOBAND includes 210,400 live-born children born in 1996–2009 on whom detailed information on prenatal exposures was obtained around gestational week 16 and 31 by telephone interviews in DNBC, and around gestational week 17 and 30 by self-administered questionnaires in MoBa, as described in more detail elsewhere [20]. We excluded children without any prenatal information from the earliest data collection (*n* = 16,691), infant deaths (*n* = 634), and children with incomplete information on maternal thyroid disorder (*n* = 157), leaving 192,918 children for the final analyses.

### Register-based data

Diagnoses of thyroid disorder in the Danish mothers were derived from the Danish National Patient Register [23], which keeps information on hospital admissions since 1977 and outpatient visits since 1995. Until 1994, all diagnoses were coded using the eighth version of the Internal Classification of Diseases (ICD-8), and since 1995, the tenth version (ICD-10). The Danish National Prescription Registry [24], established in 1995, provided the Anatomical Therapeutic Chemical (ATC) codes indicating redeemed prescriptions dispensed from Danish pharmacies. The Danish Medical Birth Registry [25] and population registers in Statistics Denmark [26] provided information on characteristics of the Danish participants, while the Medical Birth Registry of Norway [27] provided information on the Norwegian participants.

### Exposure to maternal thyroid disorder

Maternal hypothyroidism and hyperthyroidism in the register-based study population were defined by a hospital diagnosis and at least one redeemed prescription of the appropriate medication, i.e. thyroid hormone (ACT-code: H03A) for hypothyroidism and anti-thyroid medication (ACT-code: H03B) for hyperthyroidism, to enhance the validity of the identified disorders. Hypothyroidism was identified by ICD-8 codes 243.99 and 244.00–244.09, and by ICD-10 codes E00, E03.0-E03.9 and E89.0, excluding 244.02, E03.0A, E03.1B, and E03.4. Hyperthyroidism was identified by ICD-8 as 242.00–242.29 and by ICD-10 as E05.0-E05.9, excluding E05.4, E05.8A, and E05.9A. There were some exceptions; when a first-time diagnosis was recorded before the establishment of the Danish National Prescription Registry in 1995, the diagnosis code exclusively determined exposure status. Further, as the Danish National Patient Register does not keep information on diagnoses made by general practitioners, we also defined hypothyroidism by at least two redemptions of thyroid hormone prescriptions and no redemptions of anti-thyroid medication prescription, and we defined hyperthyroidism by at least two redemptions of anti-thyroid prescriptions; regardless of thyroid hormone prescriptions, in case of no record of thyroid diagnosis [28–30] (see Additional file 1: eMethod 1 for information on additional coding). We included all thyroid disorders recorded until 5 years subsequent to pregnancy until 2010 inclusive. Time of identification of thyroid disorder was defined as the day of the first diagnosis code or redeemed prescription, whichever was recorded first, and categorized into; ‘before pregnancy’, ‘during pregnancy’, and ‘within 5 years after pregnancy’.

In MOBAND, information on maternal thyroid disorder in pregnancy was based on self-reports from the earliest data collection in pregnancy, which was around 16–17 weeks of gestation. The data collected in MoBa only distinguished thyroid disorder overall and could not separate hypo- and hyperthyroidism. We used information from the second pregnancy interview of DNBC and the earliest questionnaire of MoBa to define the use of thyroid medication in pregnancy.

### Cases of cerebral palsy

Danish CP cases were identified through the Danish National Cerebral Palsy Registry that includes all children surviving the first year of life with a neuro-pediatrician validated diagnosis of CP at age five-six years [31, 32]. In 1979–1996 the register covered the eastern part of Denmark, and in 1997–2007 it was nationwide. Approximately 80% of the Norwegian cases were identified though the Cerebral Palsy Registry of Norway [33]. The remaining cases were identified in record linkage with the Norwegian Patient Registry and verified by neuro-pediatricians’ examinations of medical records [34]. We assessed all CP subtypes combined and the two major subtypes: unilateral and bilateral spastic CP.

### Covariates

We used register-based data to form the variables: the year of child’s birth, maternal age (< 25, 25–29, 30–34, ≥35 years), child’s sex (boy, girl), gestational age (≥37, < 37 weeks of gestation), and maternal diabetes (no, type 1, type 2) for both study populations. For the register-based study population, we also obtained information from administrative registers about maternal educational level (basic, intermediate, higher). In MOBAND, we used self-reported information on maternal occupational status (employed, unemployed, student, receiving benefits or pension), maternal alcohol consumption per week (0, 0.5, 1–2.5, ≥3 units), and number of cigarettes smoked per day (0, 1–9, ≥10); all reported in the earliest data collection.

### Statistical analyses

We used logistic regression to estimate odds ratios (OR) with 95% confidence intervals (95% CIs) for the relationship of CP with maternal thyroid disorder. Robust standard errors were used to take into account the potential dependency between siblings. To guide our decision about which potential confounders we should adjust for, we used Directed Acyclic Graphs [35]. The adjusted models included the child’s birth year, maternal diabetes, maternal age, and maternal socioeconomic position (education/occupation), and in addition smoking and alcohol consumption in pregnancy in the MOBAND study population. We imputed missing values of covariates by use of multiple imputations (see Additional file 1: eMethod 2).

In the register-based study population, we stratified by child’s sex and gestational age, respectively, to explore whether exposure to maternal thyroid disorder has a greater impact on risk of CP in boys and children born at term. Potential misclassification of exposure was assessed by examining the agreement of self-reported and register-based information on maternal thyroid disorder in the 90,088 Danish children included in both the register-based and MOBAND study population. We subsequently applied the calculated positive and negative agreement to adjust estimates for non-differential misclassification error by using a probabilistic approach [36] (for more details see Additional file 1: eMethod 3).

To assess the sensitivity of our findings to diagnostic errors or incomplete data, we ran several secondary analyses. In the register-based study population, we restricted caseness to hospital-diagnosed maternal thyroid disorders, and also to diagnoses and redeemed prescriptions recorded from 10 years before to 5 years after pregnancy. In both study populations, we also restricted analyses to children with complete data on covariates. Finally, we included children who died within the first year of life in the study population and assessed the relationship of maternal thyroid disorder to infant death in case infant death linked to maternal thyroid disorder (perhaps with brain damage) precluded the possibility of a CP diagnosis. All analyses were performed using StataSE 14 (64-bit).

## Results

### Characteristics of the study populations

In the register-based study population, 23,625 (1.9%) of the mothers had a thyroid disorder. Hypothyroidism was recorded in 12,929 (1.0%) and hyperthyroidism in 9943 (0.8%) of the mothers; we were unable to classify the condition in the remaining 753 (< 0.1%) mothers with thyroid disorders. In MOBAND, 3042 (1.6%) of the mothers reported a thyroid disorder in pregnancy, of whom 2229 (73.3%) reported use of thyroid medication. More than twice as many mothers in MoBa (2.1%) than in DNBC (1.0%) reported a thyroid disorder. Mothers with thyroid disorders were more likely to be older, have diabetes, and deliver preterm than mothers without the disorder. Also, mothers with thyroid disorders were more likely to have intermediate or higher education in the register-based study population, were more likely to be unemployed or receive benefits/pension, and to consume less alcohol and smoke less in pregnancy in MOBAND (Table 1).

**Table 1:** Characteristics of exposed versus unexposed in the register-based and MOBAND study population, respectively. Contains data prior to multiple imputation of missing values

	Danish register-based study population No = 1,270,079		MOBAND study population No = 192,918	
Characteristics	Exposed to maternal thyroid disorder No = 23,625	Unexposed No = 1,246,454	Exposed to maternal thyroid disorder No = 3042	Unexposed No = 189,876
	No (%)	No (%)	No (%)	No (%)
Maternal age				
≥ 30 years	13,921 (58.9)	544,852 (43.7)	1997 (65.6)	103,084 (54.3)
Missing	0 (−)	0 (−)	0 (−)	0 (−)
Maternal occupational status^a^				
Unemployed/ receiving benefits or pension	–	–	310 (10.2)	6816 (3.6)
Missing	–	–	50 (1.6)	2055 (1.1)
Maternal educational level^b^				
Basic	4781 (20.2)	280,337 (22.5)	–	–
Missing	694 (2.9)	93,414 (7.5)	–	–
Maternal diabetes				
Diabetes type 1 or 2	518 (2.2)	6362 (<1)	61 (2.0)	855 (<1)
Missing	0 (−)	0 (−)	0 (−)	0 (−)
Smoking in pregnancy				
≥ 1 cigarettes/day	–	–	293 (9.6)	23,009 (12.1)
Missing	–	–	18 (<1)	900 (<1)
Alcohol consumption in pregnancy				
≥ 0.5 units/week	–	–	529 (17.4)	50,510 (26.6)
Missing	–	–	206 (6.8)	10,129 (5.3)
Child’s sex				
Male	11,690 (49.5)	606,514 (48.7)	1572 (51.7)	97,265 (51.2)
Missing	5 (<1)	864 (<1)	0 (−)	2 (< 1)
Gestational age				
< 37 weeks (preterm)	1796 (7.6)	72,204 (5.8)	233 (7.7)	11,691 (6.2)
Missing	0 (−)	5 (< 1)	12 (< 1)	423 (<1)

*Abbreviations*: *MOBAND* MOthers and BAbies in Norway and Denmark, *No* number in complete case data

^a^Socioeconomic measure in the MOBAND study population

^b^Socioeconomic measure in the register-based study population

### Maternal thyroid disorder and risk of cerebral palsy

CP was diagnosed in 2798 children in the register-based study population. Bilateral spastic CP was the most common subtype with 1490 cases, while 912 children had unilateral spastic CP. Maternal thyroid disorder diagnosed or treated for the first time before pregnancy until 5 years subsequent to pregnancy was not associated with CP overall or either of the subtypes: unilateral or bilateral spastic CP (Table 2). Maternal thyroid disorder identified during pregnancy was associated with increased risk of unilateral spastic CP (adjusted OR 3.1 (95% CI: 1.2–8.4) (Table 2). Sufficient statistical power was unavailable to address the timing of identification of hypo- and hyperthyroidism separately. In the stratified analyses, estimates were similar across strata of sex and gestational ages, respectively, and no interaction was suggested (*p*-values for interaction > 0.2, Additional file 1: eTable 1–2), though the estimates were imprecise.

**Table 2:** Maternal thyroid disorder and risk of cerebral palsy

*Danish register-based study population*										
Maternal thyroid disorder^a^		All CP (No = 2798)			Unilateral Spastic CP (No = 912)			Bilateral Spastic CP (No = 1490)		
	No	No	OR^b^ (95% CI)	OR^c^ (95% CI)	No	OR^b^ (95% CI)	OR^c^ (95% CI)	No	OR^b^ (95% CI)	OR^c^ (95% CI)
No thyroid disorder	1,246,454	2748	1 (ref.)	1 (ref.)	894	1 (ref.)	1 (ref.)	1465	1 (ref.)	1 (ref.)
Thyroid disorder^d^	23,625	50	0.96 (0.73–1.27)	0.99 (0.74–1.31)	18	1.06 (0.67–1.69)	0.93 (0.58–1.48)	25	0.90 (0.61–1.34)	1.03 (0.69–1.53)
Hypothyroidism	12,929	26	0.91 (0.62–1.34)	0.95 (0.64–1.39)	9	0.97 (0.50–1.87)	0.83 (0.43–1.61)	13	0.86 (0.50–1.48)	1.01 (0.58–1.74)
Hyperthyroidism	9943	24	1.10 (0.73–1.64)	1.11 (0.94–1.66)	9	1.26 (0.65–2.43)	1.13 (0.59–2.18)	12	1.03 (0.58–1.81)	1.13 (0.64–1.99)
Identification of thyroid disorder										
No	1,246,454	2748	1 (ref.)	1 (ref.)	894	1 (ref.)	1 (ref.)	1465	1 (ref.)	1 (ref.)
Before pregnancy	9622	20	0.94 (0.61–1.46)	0.97 (0.62–1.50)	7	1.01 (0.48–2.13)	0.86 (0.41–1.81)	11	0.97 (0.54–1.76)	1.13 (0.62–2.04)
In pregnancy	1551	7	2.05 (0.98–4.32)	2.00 (0.95–4.21)	4	3.60 (1.35–9.63)	3.14 (1.17–8.42)	<4^f^	NE	NE
≤ 5 years after pregnancy	12,452	23	0.84 (0.56–1.26)	0.87 (0.58–1.31)	7	0.78 (0.37–1.65)	0.70 (0.33–1.48)	12	0.82 (0.46–1.45)	0.94 (0.53–1.66)
*MOBAND study population*										
Maternal thyroid disorder in pregnancy		All CP (No = 402)								
	No	No	OR^b^ (95% CI)	OR^c^ (95% CI)	OR^e^ (95% CI)					
No thyroid disorder	189,876	394	1 (ref.)	1 (Ref.)	1 (ref.)					
Thyroid disorder	3042	8	1.27 (0.63–2.55)	1.18 (0.58–2.39)	1.17 (0.58–2.38)					
No medication^g^	534	3	2.71 (0.87–8.46)	2.54 (0.81–7.94)	2.54 (0.81–7.93)					
Use of medication^g^	2229	4	0.86 (0.32–2.30)	0.82 (0.30–2.19)	0.82 (0.30–2.19)					

Multiple logistic regressions were used to calculate odds ratios (ORs) with 95% confidence intervals (95% CIs) in data with multiple imputation of missing values

*Abbreviations*: *CP* cerebral palsy, *No* number in complete case data, *NE* no estimate, *MOBAND* MOthers and BAbies in Norway and Denmark

^a^Maternal thyroid disorder identified before pregnancy until 5 years subsequent to pregnancy

^b^Unadjusted

^c^Adjusted for birth year, maternal age, maternal diabetes, and maternal socioeconomic status

^d^Unclassifiable thyroid disorder is included in the overall measure of thyroid disorder in addition to hypothyroidism and hyperthyroidism

^e^Adjusted for birth year, maternal age, maternal diabetes, maternal socioeconomic status, and smoking and alcohol consumption in pregnancy

^f^The governmental organization Statistics Denmark responsible for the register-based data, do not allow data extraction of figures below four

^g^Information on maternal use of thyroid medication was available for 2763 children exposed to maternal thyroid disorder in the MOBAND study, missing information has been imputed

In MOBAND, 402 children were diagnosed with CP of whom 47.8% had bilateral, and 37.1% had unilateral spastic CP. Six of the children with bilateral spastic CP were exposed to maternal thyroid disorder, while only one child with unilateral spastic CP was exposed, making it unfeasible to confirm or deny the register findings for unilateral spastic CP. The estimates for maternal thyroid disorder and CP overall did not suggest an association (adjusted OR 1.2 (95% CI: 0.6–2.4), Table 2), although an high, but imprecise estimate of risk for bilateral spastic CP (adjusted OR 1.9 (95% CI: 0.8–4.3)) was noted. A higher estimate of overall CP risk (adjusted OR 2.5 (95% CI: 0.8–7.9)) was seen in children exposed to an untreated thyroid disorder than to a treated disorder, but these estimates were also imprecise. Adjustment for maternal lifestyle factors did not alter the results (Table 2).

### Assessment of bias

The proportion of positive agreement between self-reported and register-based information on maternal thyroid disorder was 60%, while the proportion of negative agreement was 99%. Estimates adjusted for systematic bias were unstable but suggested that non-differential misclassification of exposure biased towards the null (Table 3). Further, changing the categorization of maternal thyroid disorder and restricting the analyses to complete cases did not alter the results. Finally, we found an increased risk of infant death in children exposed to both maternal hypothyroidism (unadjusted OR 1.4 (95% CI: 1.1–1.9)) and hyperthyroidism (unadjusted OR 1.9 (95% CI. 1.4–2.4)).

**Table 3:** Assessment of misclassification of exposure to maternal thyroid disorder

Agreement between sources of maternal thyroid disorder in 90,088 danish children included in both the danish register-based study population and MOBAND study population					
*Register-based information* ^a^	*Self-report*				
	Unexposed	Hypothyroidism	Hyperthyroidism	Unclassifiable	Total
Unexposed	**88,841**	110	48	158	89,157
Hypothyroidism	164	**240**	17	68	489
Hyperthyroidism	213	21	**147**	21	402
Unclassifiable	19	14	4	**3**	40
Total	89,237	385	216	250	90,088
	Observed proportionate agreement				
*Proportion of positive agreement*	0.60				
Proportion of negative agreement	0.99				
Kappa	0.60, *p* < 0.001				
*Bias-adjusted estimates of thyroid disorder and risk of cerebral palsy*					
	All CP	Unilateral spastic CP	Bilateral spastic CP		
Bias-adjusted OR^b^ (study error)^c^ in register-based study population	0.89 (0.40–1.21)	1.20 (0.72–3.83)	0.77 (0.24–1.22)		

Abbreviations: *CP* cerebral palsy, *OR* odds ratio

^a^The exposure window of the register-based measure was changed to an identified thyroid disorder before pregnancy until week 18 of gestation in order to correspond with the measure based on maternal self-report in MOBAND

^b^Raw estimates adjusted for misclassification using a probabilistic approach assuming non-differential misclassification of exposure. Assumptions regarding level of sensitivity and specificity were guided by the calculated positive and negative agreement (for more details see Additional file 1: eMethod 3)

^c^Study error includes both systematic error (interval encompassing 95% of the corrected estimates) and random error (95% confidence interval)

## Discussion

### Main findings

Results from the register part of this study show that thyroid disorder in pregnancy is not related to bilateral spastic CP, but may possibly be related to unilateral CP. Though the statistical power of MOBAND data was limited, information on lifestyle during pregnancy enabled us to perform more thorough control for potential confounders, which did not influence the results.

### Potential mechanisms

We studied unilateral and bilateral spastic CP separately, because they may have distinct etiological profiles. It has been hypothesized that thyroid hormone deficiency can cause CP by altering myelination, differentiation, and migration of nerve cells [8], which would likely be reflected in bilateral damage to the brain. An increased risk of bilateral spastic CP was suggested in MOBAND data only, but the estimate was unstable, and the finding was not replicated in the larger register-based study. The most striking finding in this study, a three-fold increase in risk of unilateral CP in association with thyroid disorder identified in pregnancy, is biological plausible. Maternal thyroid disorder may affect the coagulation system and increase the risk of thrombosis (leading to ischemia) and bleeding [16, 17], and such vascular events most likely cause unilateral spastic CP [37]. In support of this line of reasoning, markers of coagulation abnormalities including Factor V Leiden mutations, which implying an increased risk of thrombosis, have been linked to spastic CP [38]; especially, the unilateral subtype, though the evidence is sparse [39].

The male excess of CP indicates perhaps a heightened vulnerability to brain injury in boys [40], and abnormal thyroid hormone levels may affect boys differently than girls [41], but we were unable to find any sex differences in the association between maternal thyroid disorder and the risk of CP. Further, we hypothesized that the risk of CP in association with maternal thyroid disorder would be elevated mainly in children born at term, as we expect prenatal factors to play a greater role in the etiology of CP in children born at term than in children born preterm [40]. The estimates were unstable after stratification by gestational age, and there was no indication of any differences in risk.

### Previous findings

The syndrome of neurological cretinism provides a convincing indication of a link between maternal thyroid disturbances and CP. Children born to women with severe iodine deficiency have a substantial risk of impaired cognitive and motor function, and clinical findings and brain imaging compatible with CP has been observed in children with the neurologic form of endemic cretinism [42]. Maternal thyroid diseases have only been investigated in relation to risk of CP in few studies. Nelson et al. found in a cohort study of 45,559 children, of whom 189 had CP, an increased risk of CP in infants with a birth weight ≥ 2500 g who were born to women with hyperthyroidism and in infants exposed to maternal thyroid hormone and estrogen supplementation in pregnancy [7]. In another study of 183 children with CP and 549 controls without CP, more cases than controls were born to women who were treated with thyroid hormone; however, the difference was not statistically significant [12]. Recent register-based studies from Denmark by Andersen et al. [28, 43] have indicated that thyroid disorders identified subsequent to pregnancy, especially within 5 years after pregnancy, are correlated with increased risk of attention deficit hyperactivity disorder, autism spectrum disorder, and seizures. We found a tendency to increased risk of unilateral spastic CP in children born to women with thyroid disorders identified during pregnancy. These children will probably have been exposed to abnormal thyroid hormone levels in utero, as abnormal levels may be present for a period before the disorder is diagnosed and treated for the first time.

### Strengths and limitations

The large scale register data enabled examination of different subtypes of thyroid disorder and CP, but lacked information on maternal lifestyle factors. Although the statistical power of MOBAND was limited, among prospective cohort studies, MOBAND holds by far the largest sample of CP cases with detailed information on lifestyle collected during pregnancy, which allowed us to address such potential confounders.

CP was verified by neuropediatricians based on clinical presentation when the children were five-six years old, unaware of maternal illness during pregnancy, which enhances the validity of the CP diagnoses. Likewise, measures of maternal thyroid disorder were not affected by knowledge of whether the child had CP, because of the register recording or self-reporting during pregnancy. We restricted our analyses to children surviving to age 1 year as CP cannot reliably be diagnosed before this age. We saw an increased risk of infant death in children prenatally exposed to maternal thyroid disorders. If children with brain damage compatible with CP also are more likely to die before CP can be diagnosed, this restriction might have biased our results towards the null.

We did not have information on causes of thyroid disorders, which may be important for understanding the mechanism by which unregulated thyroid disorder is associated with risk of CP. Moreover, it is possible that autoimmune conditions confound the association. Thyroid disorders in reproductive-age women are most often autoimmune in origin, and may be associated with other autoimmune manifestations [40]. Autoimmune disorders might lead to CP either because autoantibodies are themselves pathogenic, or because of the presence of inflammation in autoimmunity, which is an established risk factor for spastic CP [44]. We adjusted for maternal diabetes, but it is possible that confounding by other autoimmune diseases and other abnormalities that coexist with thyroid disorder, for which we did not have information, had occurred.

The comparison of measures of thyroid disorder from different sources revealed that some non-differential misclassification had occurred, which may have led to an underestimation of the association of thyroid disorder and CP. In the register data, we were unable to identify women who had a thyroid disorder diagnosed outside hospital settings and who did not redeem any thyroid medication before the establishment of the Danish National Prescription in 1995. Moreover, we may have categorized some women as exposed to maternal thyroid disorder in pregnancy, even though the mother may have recovered from the disease before pregnancy, e.g. postpartum thyroiditis is often transient. In MOBAND, some women may have been unaware of their diagnoses, which also may have led to misclassification. However, in the register-based study population, we used thyroid disorder diagnosed within 5 years after pregnancy as a proxy for subclinical and asymptomatic thyroid diseases in pregnancy, since recent findings from DNBC based on blood samples drawn in early pregnancy have shown that abnormal thyroid function may be present for a period before the disorder is identified and that asymptomatic thyroid diseases are common [45]. We anticipate that much initially asymptomatic thyroid disorders would have come to medical attention within 5 years, and that the milder and most subclinical forms of thyroid disorders would be less likely to have an impact on risk of CP. Moreover, as we did not have access to information on the actual thyroid hormone level, we cannot know whether women with an identified thyroid disorder actually had abnormal thyroid hormone values during pregnancy or whether the women had a hypo- or hyperthyroid condition due to overtreatment. The natural next step is to make use of maternal blood samples collected during pregnancy to study the link between maternal thyroid disorder and CP.

## Conclusion

It is reassuring that maternal thyroid disorders do not seem to be related to the predominant CP subtype, bilateral spastic CP. However, our findings hint that risk of unilateral spastic CP may be higher in children of mothers with thyroid disorder identified in pregnancy, which presumably is more unregulated than disorders identified before pregnancy, since abnormal thyroid hormone levels may be present for a period before the disorder is diagnosed and treated for the first time. We cannot exclude the possibility that the observed association is due to chance or unmeasured confounding. Replication of our findings in studies that test maternal thyroid hormone level in early pregnancy is therefore needed.

## Additional file

Additional file 1: The file contain supplementary methods and tables. (DOCX 32 kb)

## Abbreviations

95% CI: 95% confidence interval
CP: Cerebral palsy
DNBC: The Danish National Birth Cohort
MoBa: The Norwegian Mother and Child Cohort study
MOBAND: The MOthers and BAbies in Norway and Denmark
OR: Odds ratio

## References

[1] Oskoui M, Coutinho F, Dykeman J, Jette N, Pringsheim T (2013). An update on the prevalence of cerebral palsy: a systematic review and meta-analysis. Dev Med Child Neurol.

[2] Nelson KB, Blair E (2015). Prenatal factors in singletons with cerebral palsy born at or near term. N Engl J Med.

[3] Haddow JE, Palomaki GE, Allan WC, Williams JR, Knight GJ, Gagnon J, O'Heir CE, Mitchell ML, Hermos RJ, Waisbren SE (1999). Maternal thyroid deficiency during pregnancy and subsequent neuropsychological development of the child. N Engl J Med.

[4] Klein RZ, Mitchell ML (2002). Maternal hypothyroidism and cognitive development of the offspring. Curr Opin Pediatr.

[5] Pop VJ, Brouwers EP, Vader HL, Vulsma T, van Baar AL, de Vijlder JJ (2003). Maternal hypothyroxinaemia during early pregnancy and subsequent child development: a 3-year follow-up study. Clin Endocrinol.

[6] Pop VJ, Kuijpens JL, van Baar AL, Verkerk G, van Son MM, de Vijlder JJ, Vulsma T, Wiersinga WM, Drexhage HA, Vader HL (1999). Low maternal free thyroxine concentrations during early pregnancy are associated with impaired psychomotor development in infancy. Clin Endocrinol.

[7] Nelson KB, Ellenberg JH (1986). Antecedents of cerebral palsy. Multivariate analysis of risk. N Engl J Med.

[8] Hong T, Paneth N (2008). Maternal and infant thyroid disorders and cerebral palsy. Semin Perinatol.

[9] Leviton A, Paneth N, Reuss ML, Susser M, Allred EN, Dammann O, Kuban K, Van Marter LJ, Pagano M (1999). Hypothyroxinemia of prematurity and the risk of cerebral white matter damage. J Pediatr.

[10] Reuss ML, Paneth N, Pinto-Martin JA, Lorenz JM, Susser M (1996). The relation of transient hypothyroxinemia in preterm infants to neurologic development at two years of age. N Engl J Med.

[11] Suzumura H, Nitta A, Tsuboi Y, Watabe Y, Kuribayashi R, Arisaka O (2011). Thyroxine for transient hypothyroxinemia and cerebral palsy in extremely preterm infants. Pediatr Int.

[12] Blair E, Stanley F (1993). When can cerebral palsy be prevented? The generation of causal hypotheses by multivariate analysis of a case-control study. Paediatr Perinat Epidemiol.

[13] Jain V, Agarwal R, Deorari AK, Paul VK (2008). Congenital hypothyroidism. Indian J Pediatr.

[14] Burrow GN, Fisher DA, Larsen PR (1994). Maternal and fetal thyroid function. N Engl J Med.

[15] Hung PL, Huang CC, Huang HM, Tu DG, Chang YC (2013). Thyroxin treatment protects against white matter injury in the immature brain via brain-derived neurotrophic factor. Stroke.

[16] Stuijver DJ, van Zaane B, Romualdi E, Brandjes DP, Gerdes VE, Squizzato A (2012). The effect of hyperthyroidism on procoagulant, anticoagulant and fibrinolytic factors: a systematic review and meta-analysis. Thromb Haemost.

[17] Squizzato A, Romualdi E, Buller HR, Gerdes VE (2007). Clinical review: thyroid dysfunction and effects on coagulation and fibrinolysis: a systematic review. J Clin Endocrinol Metab.

[18] Laurberg P, Andersen SL, Hindersson P, Nohr EA, Olsen J (2016). Dynamics and predictors of serum TSH and fT4 reference limits in early pregnancy: a study within the Danish National Birth Cohort. J Clin Endocrinol Metab.

[19] Pedersen CB, Gotzsche H, Moller JO, Mortensen PB (2006). The Danish Civil Registration System. A cohort of eight million persons. Dan Med Bull.

[20] Tollanes MC, Strandberg-Larsen K, Forthun I, Petersen TG, Moster D, Andersen AM, Stoltenberg C, Olsen J, Wilcox AJ (2016). Cohort profile: cerebral palsy in the Norwegian and Danish birth cohorts (MOBAND-CP). BMJ Open.

[21] Olsen J, Melbye M, Olsen SF, Sorensen TI, Aaby P, Andersen AM, Taxbol D, Hansen KD, Juhl M, Schow TB (2001). The Danish National Birth Cohort--its background, structure and aim. Scand J Public Health.

[22] Magnus P, Birke C, Vejrup K, Haugan A, Alsaker E, Daltveit AK, Handal M, Haugen M, Hoiseth G, Knudsen GP (2016). Cohort profile update: the Norwegian mother and child cohort study (MoBa). Int J Epidemiol.

[23] Lynge E, Sandegaard JL, Rebolj M (2011). The Danish National Patient Register. Scand J Public Health.

[24] Kildemoes HW, Sorensen HT, Hallas J (2011). The Danish National Prescription Registry. Scand J Public Health.

[25] Knudsen LB, Olsen J (1998). The Danish medical birth registry. Dan Med Bull.

[26] Mortensen LH, Helweg-Larsen K, Andersen AM (2011). Socioeconomic differences in perinatal health and disease. Scand J Public Health.

[27] Irgens LM (2000). The Medical Birth Registry of Norway. Epidemiological research and surveillance throughout 30 years. Acta Obstet Gynecol Scand.

[28] Andersen S, Laurberg P, Wu C, Olsen J (2014). Attention deficit hyperactivity disorder and autism spectrum disorder in children born to mothers with thyroid dysfunction: a Danish nationwide cohort study. Bjog.

[29] Andersen SL, Olsen J, Wu CS, Laurberg P (2014). Smoking reduces the risk of hypothyroidism and increases the risk of hyperthyroidism: evidence from 450,842 mothers giving birth in Denmark. Clin Endocrinol.

[30] Andersen SL, Olsen J, Wu CS, Laurberg P (2014). Psychiatric disease in late adolescence and young adulthood. Foetal programming by maternal hypothyroidism?. Clin Endocrinol (Oxf).

[31] Topp M, Langhoff-Roos J, Uldall P (1997). Validation of a cerebral palsy register. J Clin Epidemiol.

[32] Uldall P, Michelsen SI, Topp M, Madsen M (2001). The Danish Cerebral Palsy Registry. A registry on a specific impairment. Dan Med Bull.

[33] Andersen GL, Irgens LM, Haagaas I, Skranes JS, Meberg AE, Vik T (2008). Cerebral palsy in Norway: prevalence, subtypes and severity. Eur J Paediatr Neurol.

[34] Hollung SJ, Vik T, Wiik R, Bakken IJ, Andersen GL. Completeness and correctness of cerebral palsy diagnoses in two health registers: implications for estimating prevalence. Dev Med Child Neurol. 2016;59:402–6. 10.1111/dmcn.13341 27896812

[35] Hernan MA, Hernandez-Diaz S, Werler MM, Mitchell AA (2002). Causal knowledge as a prerequisite for confounding evaluation: an application to birth defects epidemiology. Am J Epidemiol.

[36] Lash TL, Fox MP, Fink AK. A Guide to Implementing Quantitative Bias Analysis. In: Applying Quantitative Bias Analysis to Epidemiologic Data. 1st ed. London, New York: Springer; 2009. p. 13–32. https://link.springer.com/content/pdf/10.1007%2F978-0-387-87959-8.pdf.

[37] Kirton A, deVeber G (2006). Cerebral palsy secondary to perinatal ischemic stroke. Clin Perinatol.

[38] Nelson KB, Dambrosia JM, Grether JK, Phillips TM (1998). Neonatal cytokines and coagulation factors in children with cerebral palsy. Ann Neurol.

[39] Thorarensen O, Ryan S, Hunter J, Younkin DP (1997). Factor V Leiden mutation: an unrecognized cause of hemiplegic cerebral palsy, neonatal stroke, and placental thrombosis. Ann Neurol.

[40] Himmelmann K, Hagberg G, Beckung E, Hagberg B, Uvebrant P (2005). The changing panorama of cerebral palsy in Sweden. IX. Prevalence and origin in the birth-year period 1995-1998. Acta Paediatr.

[41] Briet JM, van Wassenaer AG, van Baar A, Dekker FW, Kok JH (1999). Evaluation of the effect of thyroxine supplementation on behavioural outcome in very preterm infants. Dev Med Child Neurol.

[42] Ma T, Lian ZC, Qi SP, Heinz ER, DeLong GR (1993). Magnetic resonance imaging of brain and the neuromotor disorder in endemic cretinism. Ann Neurol.

[43] Andersen SL, Laurberg P, Wu CS, Olsen J (2013). Maternal thyroid dysfunction and risk of seizure in the child: a Danish nationwide cohort study. J Pregnancy.

[44] Lisovska N, Daribayev Z, Lisovskyy Y, Kussainova K, Austin L, Bulekbayeva S (2016). Pathogenesis of cerebral palsy through the prism of immune regulation of nervous tissue homeostasis: literature review. Childs Nerv Syst.

[45] Andersen SL, Olsen J (2017). Early pregnancy thyroid function test abnormalities in biobank sera from women clinically diagnosed with thyroid dysfunction before or after pregnancy. Thyroid.

