# Supplementary material for: Maternal thyroid disorder in pregnancy and risk of cerebral palsy in the child: a population-based cohort study
Source: BMC Pediatr. 2018 May 31;18:181. doi: 10.1186/s12887-018-1152-5 (PMC5977482; doi:10.1186/s12887-018-1152-5)
Supplement: Supplementary file 1 — The file contain supplementary methods and tables. (DOCX 32 kb) [file 12887_2018_1152_MOESM1_ESM.docx]

ADDITIONAL FILE 1

Content:

eMethod 1. Additional coding of maternal thyroid disorder in the register based study population

eMethod 2. Multiple imputation of missing values

eMothod 3. Quantitative bias analysis of exposure misclassification

eTable 1. Maternal thyroid disorder and risk of cerebral palsy according to child's sex – Register based study population

eTable 2. Maternal thyroid disorder and risk of cerebral palsy according to gestational age – Register based study population

**eMethod 1. Additional coding of maternal thyroid disorder in the register based study population**

- If the condition converts from hyperthyroid to hypothyroidism or vice versa, the women are categorized according to the conditions they have closest to pregnancy.
- Women who both have a diagnosis of hyperthyroidism and hypothyroidism recorded in pregnancy are categorized unclassifiable.
- Women with at least two redemptions of thyroid hormone and one hyperthyroidism diagnosis, but no redemption of anti-thyroid hormone or hypothyroidism diagnosis are categorized unclassifiable.
- Women with at least two redemptions of anti-thyroid medication and one hypothyroidism diagnosis, but no redemption of thyroid hormone or hyperthyroidism diagnosis are categorized unclassifiable.

**eMethod 2. Multiple imputation of missing values**

In both study populations, multiple imputation of missing data on covariates has been performed by Chained Equations as the missing patterns are non-monotom. We included the exposure and outcome of interest, all factors that may predict the values of the missing data, and all relevant interactions in the models.

The following variables were included in the model in register based data: interaction between thyroid disorder and child's sex, interaction between thyroid disorder and gestational age, previous pregnancies, maternal education, cerebral palsy, birth year, maternal age, residence, plurality, diabetes, thyroid disorder.

The following variables were included in the model in MOBAND data: interaction between thyroid disorder and child's sex, interaction between thyroid disorder and gestational age, previous pregnancies, smoking, alcohol, apgar score, maternal occupational status, cohort affiliation, birth year, cerebral palsy, maternal age, diabetes, thyroid disorder, ivf treatment, arthritis, plurality, birth order.

**eMethod 3. Quantitative bias analysis of exposure misclassification**

We calculated observed positive and negative agreement relative to self-reported information on thyroid disorder in pregnancy and register based information on thyroid disorder in pregnancy. Information on agreement of the two measures was drawn from a 2x2 table:

| Self-report | Register based information | | |
| --- | --- | --- | --- |
|  | + (Thyroid disorder) | - (No thyroid disorder) | *Total* |
| + (Thyroid disorder) | a | b | a+b |
| - (No thyroid disorder) | c | c | c+d |
| *Total* | b+b | b+d | N |

With binary measures, positive agreement (PA) and negative agreement (NA) were calculated using the equations:

$PA=\frac{2a}{2a+b+c}$ $NA=\frac{2d}{2d+b+c}$

We computed estimates corrected for misclassification by applying a probabilistic approach. Due to lack of a golden standard, assumptions in regard of limits for sensitivity and specificity were guided by the observed positive and negative agreement. The corrected estimates were calculated based on the following assumptions:

Trapezoidal probability distribution for sensitivity with a minimum of 0.50, maximum of 0.72, and interval of equally probable values 0.56-0.66. A uniform probability distribution for specificity with a minimum of 0.98 and a maximum of maximum 0.999.

**eTable 1. Maternal thyroid disorder and risk of cerebral palsy according to child's sex – Register based study population**

| **MATERNAL THYROID DISORDER** | **CEREBRAL PALSY** | | | | | | |
| --- | --- | --- | --- | --- | --- | --- | --- |
|  | **Boys (n=651 006)** | | | **Girls (n=618 204)** | | | **Test for interaction** |
|  | **n** | **n with CP** | **OR^a^ (95% CI)** | **n** | **n with CP** | **OR^a^ (95% CI)** | **p-value** |
|  | **All CP** | | | **All CP** | | |  |
| No thyroid disorder  Thyroid disorder  Hypothyroidism  Hyperthyroidism | 639 076  11 930  6569  4969 | 1621  32  18  14 | 1 (ref.)  1.10 (0.77-1.56)  1.13 (0.71-1.80)  1.13 (0.67-1.92) | 606 514  11 690  6356  4973 | 1127  18  8  10 | 1 (ref.)  0.85 (0.53-1.35)  0.69 (0.34-1.39)  1.09 (0.58-2.04) | 0.42 |
|  |  |  |  |  |  |  | 0.23 |
|  | **Unilateral spastic CP** | | | **Unilateral spastic CP** | | |  |
| No thyroid disorder  Thyroid disorder  Hypothyroidism  Hyperthyroidism |  | 527  11  5  6 | 1 (ref.)  0.98 (0.54-1.78)  0.80 (0.34-1.92)  1.32 (0.59-2.94) |  | 367  7  4  3 | 1 (ref.)  0.87 (0.41-1.84)  0.89 (0.33-2.40)  0.90 (0.23-2.79) | 0.90 |
|  |  |  |  |  |  |  | 0.81 |
|  | **Bilateral spastic CP** | | | **Bilateral spastic CP** | | |  |
| No thyroid disorder  Thyroid disorder  Hypothyroidism  Hyperthyroidism |  | 862  17  9  8 | 1 (ref.)  1.22 (0.75-1.98)  1.21 (0.62-2.34)  1.32 (0.66-2.65) |  | 603  8  4  4 | 1 (ref.)  0.77(0.38-1.56)  0.73 (0.27-1.95)  0.88 (0.33-2.36) | 0.83 |
|  |  |  |  |  |  |  | 0.79 |
| Abbreviations: CP, cerebral palsy. n, number in complete case data.  Estimates are presented as odds ratios (ORs) with 95% confidence intervals (95% CIs). Multiple imputation of missing values are performed.  ^a^Multiple logistic regression adjusted for birth year, maternal age, maternal diabetes, and maternal socioeconomic status. | | | | | | | |

**eTable 2. Maternal thyroid disorder and risk of cerebral palsy according to gestational age – Register based study population**

| **MATERNAL THYROID DISORDER** | **SPASTIC CEREBRAL PALSY** | | | | | | |
| --- | --- | --- | --- | --- | --- | --- | --- |
|  | **Gestational week ≥ 37 (n=1 196 074)** | | | **Gestational week < 37 (n=74 000)** | | | **Test for interaction** |
|  | **n** | **n with CP** | **OR^a^ (95% CI)** | **n** | **n with CP** | **OR^a^ (95% CI)** | **p-value** |
|  | **All CP** | | | **All CP** | | |  |
| No thyroid disorder  Thyroid disorder  Hypothyroidism  Hyperthyroidism | 1 174 245  21 829  12 019  9126 | 1671  31  17  14 | 1 (ref.)  1.10 (0.70-1.45)  1.01 (0.63-1.64)  1.08 (0.64-1.83) | 71 470  2 530  1407  1041 | 1077  19  9  10 | 1 (ref.)  0.85 (0.53-1.34)  0.83 (0.43-1.60)  0.93 (0.50-1.74) | 0.20 |
|  |  |  |  |  |  |  | 0.35 |
|  | **Unilateral spastic CP** | | | **Unilateral spastic CP** | | |  |
| No thyroid disorder  Thyroid disorder  Hypothyroidism  Hyperthyroidism |  | 619  13  7  6 | 1 (ref.)  1.00 (0.58-1.74)  0.97 (0.46-2.04)  1.13 (0.51-2.53) |  | 275  5  2  3 | 1 (ref.)  0.70 (0.29-1.71)  0.55 (1.34-2.22)  0.94 (0.30-2.93) | 0.39 |
|  |  |  |  |  |  |  | 0.52 |
|  | **Bilateral spastic CP** | | | **Bilateral spastic CP** | | |  |
| No thyroid disorder  Thyroid disorder  Hypothyroidism  Hyperthyroidism |  | 764  126  6  6 | 1 (ref.)  0.96 (0.54-1.71)  0.90 (0.40-2.01)  1.11 (0.49-2.58) |  | 701  13  7  6 | 1 (ref.)  0.97 (0.56-1.70)  1.10 (0.52-2.34)  0.92 (0.41-2.05) | 0.67 |
|  |  |  |  |  |  |  | 0.77 |
| Abbreviations: CP, cerebral palsy. n, number in complete case data.  Estimates are presented as odds ratios (ORs) with 95% confidence intervals (95% CIs). Multiple imputation of missing values are performed.  ^a^Multiple logistic regression adjusted for birth year, maternal age, maternal diabetes, and maternal socioeconomic status. | | | | | | | |
